# Triple Procedure Long-Term Outcomes: Comparative Analysis of Penetrating Keratoplasty vs. DSAEK Combined with Cataract Surgery

**DOI:** 10.3390/jcm14165670

**Published:** 2025-08-11

**Authors:** Dominika Szkodny, Adam Wylęgała, Agnieszka Szkaradek, Magdalena Kijonka, Magdalena Nandzik, Edward Wylęgała

**Affiliations:** 1 Department of Ophthalmology, District Railway Hospital in Katowice, Medical University of Silesia, 40-760 Katowice, Poland; adam.wylegala@gmail.com (A.W.); kijek303@gmail.com (M.K.); nandzik.magda@gmail.com (M.N.); wylegala@gmail.com (E.W.); 2 Experimental Ophthalmology Unit, Department of Biophysics, Silesian Medical University, Panewnicka 65, 40-760 Katowice, Poland; 3 Department of Ophthalmology, Faculty of Medical Sciences, Zabrze, Medical University of Silesia, 40-760 Katowice, Poland; aszkaradekfuf@gmail.com

**Keywords:** corneal transplantation, triple procedure, penetrating keratoplasty, posterior lamellar keratoplasty, DSAEK, cataract surgery, visual rehabilitation, graft survival, refractive outcomes

## Abstract

**Background/Objectives**: This study assessed outcomes between penetrating keratoplasty (PK) and Descemet’s stripping automated endothelial keratoplasty (DSAEK) when combined with cataract surgery as part of the triple procedure. **Methods**: Retrospective analysis of 727 triple procedures (525 PK and 202 DSAEK) from 2007–2023. Graft survival, visual acuity, and refractive outcomes were analyzed. Kaplan–Meier and Cox regression were used for survival and prognostic analysis. **Results**: No statistically significant difference in survival was found (PK—42 months; DSAEK—47 months). DSAEK had better visual acuity improvement and refractive stability. PK had higher astigmatism and variability in refractive error. **Conclusions**: While graft survival was comparable, DSAEK offers superior visual rehabilitation, supporting its use when refractive predictability is important.

## 1. Introduction

Triple procedures—combining corneal transplantation and cataract extraction—are commonly employed to address coexisting corneal pathology and cataract, reducing the need for multiple surgeries and accelerating visual rehabilitation. These procedures may involve full-thickness corneal replacement through penetrating keratoplasty (PK) or selective endothelial techniques such as Descemet’s stripping automated endothelial keratoplasty (DSAEK) and Descemet membrane endothelial keratoplasty (DMEK), which were developed to minimize tissue disruption and promote faster recovery [1,2,3,4]. Our center commenced performing posterior lamellar keratoplasty in 2004, becoming the first institution in Poland to introduce this procedure [5]. Compared to PK, lamellar keratoplasty offers several advantages, including faster visual recovery, reduced risk of graft rejection, and improved structural integrity of the eye [6,7].

When combined with cataract surgery, both PK and DSAEK, or DMEK, present distinct indications and benefits [6]. Cataract extraction can be performed sequentially or simultaneously with keratoplasty—the latter is referred to as a triple procedure. Performing both surgeries simultaneously reduces overall surgical risk, anesthesia exposure, and enables a single recovery period, leading to faster rehabilitation and return to daily activities [8,9,10].

However, one limitation of the triple procedure—particularly when involving PK—is refractive unpredictability, including high ametropia and irregular astigmatism [7]. Accurate intraocular lens power calculation depends on stable and reproducible biometric data, such as corneal curvature, anterior chamber depth, and axial length. These parameters may be significantly altered after corneal transplantation, especially with full-thickness grafts, whereas lamellar procedures better preserve corneal geometry and improve refractive predictability [11].

This study evaluates the long-term outcomes of triple procedures involving PK and DSAEK, focusing on graft survival, visual acuity, and refractive performance.

## 2. Materials and Methods

This retrospective observational study reviewed hospital records of patients who underwent PK or DSAEK combined with cataract surgery at the Department of Ophthalmology, District Railway Hospital in Katowice, Poland. From January 2007 to December 2023, a total of 2816 keratoplasty procedures (penetrating and lamellar) were performed at our institution. Among these, 987 were triple procedures combining corneal transplantation and cataract extraction. Assignment to DSAEK or PK was based on clinical indications determined by the operating surgeon. Descemet stripping automated endothelial keratoplasty (DSAEK), involving donor lenticule preparation with a microkeratome, was the preferred lamellar technique for cases of isolated endothelial dysfunction with a clear corneal stroma. In contrast, PK was chosen for eyes with stromal scarring, shallow anterior chamber, prior graft failure, or other complex anterior segment pathology. Six surgeons were involved in this study, with the majority of cases being performed by three senior surgeons experienced in keratoplasty, all of whom had been performing lamellar procedures since 2004. A small number of cases were performed by two junior surgeons, and for these, the learning curve may have had a limited impact. The inclusion criteria encompassed the following: age > 18 years, corneal disease and cataract as the primary cause of visual impairment, having undergone a combined corneal transplant and cataract surgery, the availability of collected data in hospital records. A total of 727 cases fulfilled the inclusion criteria—defined as a combined procedure with available follow-up data for the duration of the study period—and were included in the analysis. The remaining cases were excluded due to loss to follow-up. The collected data included patients’ age and sex, date of surgery, surgical indication, corneal transplant technique, operating surgeon, preoperative visual acuity, concomitant conditions, number of previous corneal transplants, presence of glaucoma (pre- and post-keratoplasty), postoperative visual acuity, refractive outcomes (spherical equivalent (SE) and astigmatism), pre- and postoperative corneal topography (anterior, posterior, and total keratometry values: Ks, Kf, and astigmatism), occurrence of complications, surgical interventions following keratoplasty, donor endothelial cell count, and the method of corneal tissue retrieval. Central corneal thickness (CCT) and postoperative endothelial cell density (ECD) were assessed at 12 months postoperatively. Corneal topography was performed with the SS-OCT CASIA 2 built-in software (Version 4C.4; Corporation Tomey, Nagoya, Japan). Prior to analysis, all data were fully de-identified in compliance with institutional and ethical guidelines. All direct and indirect identifiers (e.g., names, addresses, and contact information) were removed, ensuring that no identifiable information remained in the dataset. The data were collected from 27 October 2021 to 10 January 2025. Graft survival was calculated from the date of surgery until the occurrence of graft failure. Grafts lost due to trauma were not included in the analysis. Ethical review by the Institutional Review Board of the Medical University of Silesia and approval were waived for this study due to the retrospective nature of the study and the local laws. Due to the retrospective nature of the study, a formal waiver of informed consent was granted by the Institutional Review Board. Group 1 included patients who underwent PK with cataract surgery, while Group 2 comprised those who underwent DSAEK with cataract surgery. These group designations are used consistently throughout the analysis. To ensure statistical independence, only one eye per patient was included in the final dataset. In cases where both eyes met the inclusion criteria, the eye with the more complete follow-up data was selected. All PK procedures used mechanical trephination. IOL power was calculated using preoperative biometry adjusted for post-keratoplasty topography, typically assuming a mean keratometric value of 43.0 diopters. For DSAEK cases, surgeons used actual measured keratometry. In both groups, the refractive target was mild myopia, typically between −0.5 and −1.0 diopters. The primary outcome of this study was graft survival, which was analyzed using Kaplan–Meier survival estimates. The analysis included both overall survival and subgroup analyses based on gender, transplant type (penetrating vs. DSAEK), and the presence of specific ocular and systemic comorbidities. Secondary outcomes included postoperative changes in best-corrected visual acuity (BCVA), refractive outcomes—specifically SE and astigmatism, pre- and postoperative corneal topography, and the occurrence of postoperative complications. Graft failure was defined as the irreversible loss of corneal clarity resulting from either immune rejection or endothelial decompensation. Rejection was categorized as endothelial if associated with visible signs of inflammation, such as keratic precipitates or anterior chamber reaction. However, mild rejection episodes that responded fully to medical treatment and did not result in sustained corneal opacification were not considered graft failures. Decompensation was diagnosed clinically based on persistent corneal edema or stromal haze unresponsive to therapy. Grafts that failed due to trauma or unrelated ocular pathology were excluded from the failure analysis.

Descriptive statistics were employed to summarize patient demographics and clinical characteristics. Cox regression models were used to estimate hazard ratios (HR) and assess factors influencing graft survival. Statistical significance was defined as *p* < 0.05. Univariate and multivariate Cox regression analyses were performed to identify factors influencing graft survival, including ocular comorbidities, general comorbidities, patient age, and K1 anterior keratometry values. To address indication bias a propensity score matching (PSM) was performed using Statistica v 12 (Statsoft, Palo Alto, CA, USA).

Jamovi v2.6 was used to conduct statistical analysis [12,13,14,15,16,17,18,19,20,21,22].

## 3. Results

### 3.1. Demographic and Clinical Characteristics

A total of 727 patients were included in the analysis. The gender distribution showed that 57.1% (*n* = 415) of the cohort were female, while 42.9% (*n* = 312) were male. The median age of the participants was 68 years (IQR: 21–95), with a standard deviation of 12.9 years.

Among the patients, 40.1% were diagnosed with Fuchs endothelial cell dystrophy, making it the most prevalent condition. Ocular involvement was nearly evenly distributed, with 49.5% of cases affecting the right eye and 50.5% affecting the left eye. Regarding transplantation type, 72.1% of patients underwent PK, while 27.9% received DSAEK.

The analysis of regraft frequency indicated that 85.4% of patients did not require a regraft, while 13.6% underwent a single regraft, and only 0.6% required two or more regrafts. Postoperatively, glaucoma was identified in 36.3% of cases, whereas the remaining 63.7% did not develop glaucoma. Surgeon involvement varied, with the most frequent category performing 37.4% of procedures.

### 3.2. Tissue Characteristics and Rejection Rates

Regarding tissue procurement, 75.2% of tissues were obtained from multiple organ donors or autopsies, while the remaining samples were acquired from other sources. Rejection and decompensation rates were low, with 86.7% of patients experiencing no rejection episodes, 3.3% experiencing a single episode, and less than 10% having multiple rejection events. Rejection rates varied among the different types of transplants: 1.5% lamellar and 7.8% penetrating.

### 3.3. Survival and Outcome Measures

**Figure 1:**
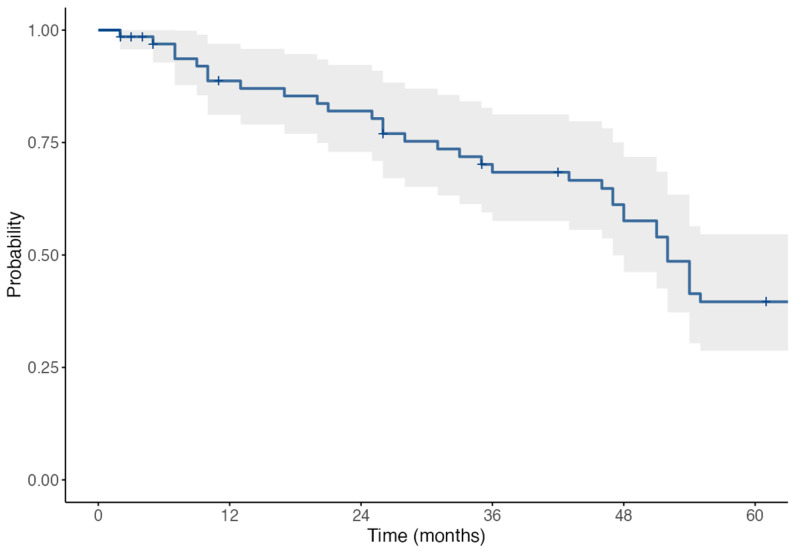
Kaplan–Meier survival curve + sign displays the censored cases.

The Kaplan–Meier survival analysis was performed to assess the cumulative incidence of graft rejection events over a 60-month follow-up period. A mean time to rejection of 45.8 months, with a median of 42.5 months ± 36.8. By 60 months, 75% of rejections had occurred (Figure 1).

Due to the 2007–2023 study span, not all patients reached the full 60-month follow-up. However, the number of patients at risk at each key timepoint is provided within the Kaplan–Meier analysis. The sample includes a range of follow-up durations, and censoring was applied accordingly to patients with shorter observation periods. This explains the gradual decline in patients contributing to survival estimates over time.

### 3.4. Survival Analysis by Transplant Type

The median survival times were analyzed for two groups of transplant types: penetrating transplant with cataract (Group 1) and DSAEK with cataract (Group 2):

For DSAEK with cataract, the median survival time was 47 months (95% CI: 26–51).

For penetrating transplant with cataract, the median survival time was slightly shorter, at 42 months (95% CI: 26–52).

**Figure 2:**
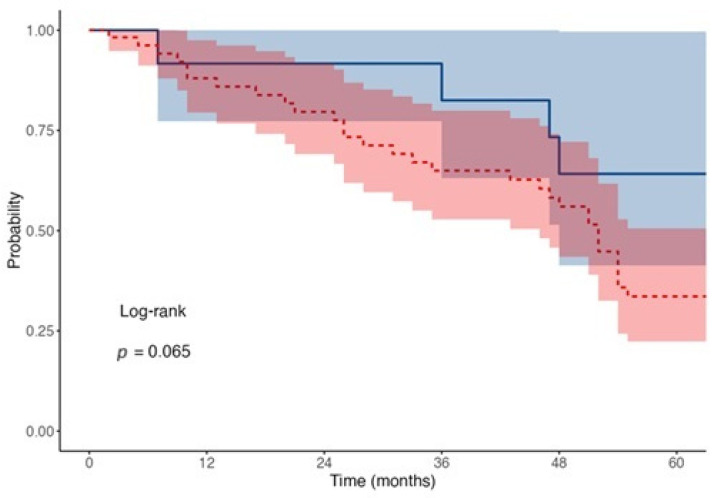
Kaplan–Meier survival curves for transplant type. Red dotted line shows the rejection events for penetrating while blue solid for lamellar transplants.

The median survival time represents the point at which 50% of patients in each group experienced the event, indicating that half of the patients survived beyond these time frames. There was no significant difference in survival risk between the two groups HR of 0.97 (95% CI: 0.44–2.15, *p* = 0.941) (Figure 2).

The mean follow-up duration was 38.2 ± 22.5 months for PK and 34.7 ± 19.8 months for DSAEK. At each Kaplan–Meier timepoint (12, 36, and 60 months), the number of patients at risk was as follows: DSAEK—6, 5, 2; PK—39, 27, 14, respectively.

### 3.5. Survival Probability at 1, 3, and 5 Years

Survival probabilities were analyzed at key intervals (12, 36, and 60 months) for both transplant types:

DSAEK with Cataract:

- At 12 months, survival probability was 85.7% (95% CI: 63.3–100.0%), with 6 subjects at risk and 1 event recorded.
- At 36 months, survival probability dropped to 57.1% (95% CI: 30.1–100.0%), with 5 subjects at risk and 2 events recorded.
- At 60 months, survival probability further decreased to 28.6% (95% CI: 8.9–92.2%), with 2 subjects at risk and 2 events recorded.

Penetrating Transplant with Cataract:

- At 12 months, survival probability was 73.6% (95% CI: 62.6–86.5%), with 39 subjects at risk and 14 events recorded.
- At 36 months, survival probability decreased to 50.9% (95% CI: 39.1–66.3%), with 27 subjects at risk and 12 events recorded.
- At 60 months, survival probability was 26.4% (95% CI: 16.9–41.4%), with 14 subjects at risk and 13 events recorded.

Among patients with Fuchs’ dystrophy (*n* = 292), the 5-year survival rate was 61.2%, versus 48.5% in other etiologies. Although not statistically significant, this may reflect differences in underlying pathology.

### 3.6. Cox Regression Analysis

The univariable Cox regression analysis for the rejection and decompensation of transplants identified significant factors influencing survival. K2 real before was a notable factor, with a hazard ratio (HR) of 1.05 (95% CI: 1.00–1.11, *p* = 0.060), suggesting a trend toward significance, although it did not reach the traditional cutoff for statistical significance. Similarly, posterior astigmatism before showed an HR of 2.14 (95% CI: 1.25–3.66, *p* = 0.006), indicating a detrimental effect on survival.

Ocular comorbidities, such as glaucoma, AMD, high Myopia, post uveitic and post herpetic, did not show statistically significant associations, with *p*-values of >0.1. For general comorbidities, neither of the studied diseases showed any significance.

A multivariable Cox regression analysis was conducted to evaluate potential factors influencing transplant survival. The variables included K2 real before the procedure and posterior corneal astigmatism before. Overall, posterior corneal astigmatism before showed HR of 2.01 (1.15–3.51, *p* = 0.014).

### 3.7. Visual Acuity and Refractive Error

Preoperative visual acuity was worse in PK (Group 1) compared to DSAEK (Group 2). The mean preoperative visual acuity in Group 1 was 0.232 ± 0.123, whereas in Group 2, it was 0.290 ± 0.138. After surgery, both groups showed significant improvement in best-corrected visual acuity—Group 2: 0.505 ± 0.200, compared to 0.372 ± 0.150 in Group 1.

Group 1 had a mean SE of 1.83 (±4.39), while Group 2 showed a lower mean of 1.02 (±1.24). The median SE values were 1.50 and 1.00, respectively, indicating a slight difference in central tendency. The standard deviation in Group 1 (4.39) was notably larger than in Group 2 (1.24), suggesting greater variability.

Astigmatism followed a similar trend, with Group 1 averaging 3.17 (±2.02) and Group 2 1.42 (±1.05). The median values of 3.00 and 1.50, respectively, further highlight this difference. Again, Group 1 exhibited greater variability, as reflected in its higher standard deviation (2.02 vs. 1.05). These findings suggest that Group 1 experienced significantly higher and more variable refractive error postoperatively.

The results of the *t*-test indicated a statistically significant difference in SE values between Group 1 and Group 2 (*p* = 0.026). A statistical comparison of astigmatism values between the groups showed a highly significant difference (*p* < 0.0001) (Table 1).

### 3.8. Central Corneal Thickness (CCT)

No significant differences were observed in CCT between Group 1 and Group 2 before or after transplantation. Preoperative mean CCT was 647.97 µm in Group 2 and 705.79 µm in Group 1 (*p* = 0.126). Postoperatively, CCT remained comparable (Group 2—604.77 µm; Group 1—632.63 µm, *p* = 0.210).

### 3.9. Corneal Astigmatism (CASIA)

Anterior corneal astigmatism was significantly higher in Group 1, preoperatively (6.51 D vs. 1.59 D, *p* < 0.001) and postoperatively (7.21 D vs. 1.57 D, *p* < 0.001). Similar trends were observed for posterior astigmatism (preop—1.21 D vs. 0.63 D, *p* = 0.002; postop—1.65 D vs. 0.88 D, *p* < 0.001). Total corneal astigmatism remained significantly greater in Group 1 both before and after surgery (*p* < 0.001).

### 3.10. Corneal Curvature (K1, K2, Kmax)

Preoperatively, Group 1 exhibited significantly steeper anterior K1 (47.07 D vs. 43.89 D, *p* = 0.048) and anterior K2 (53.77 D vs. 45.28 D, *p* < 0.001). Postoperatively, Group 1 showed a greater reduction in K1 (39.05 D vs. 42.33 D, *p* = 0.033), while posterior K2 remained significantly different (*p* = 0.002). Maximum keratometry (Kmax) was significantly higher in Group 1 both before (64.76 D vs. 47.88 D, *p* < 0.001) and after transplantation (64.13 D vs. 45.90 D, *p* < 0.001), (Table 2).

### 3.11. Endothelial Cell Density (ECD)

A total of 511 observations were included in the pre-intervention group, and 232 observations in the post-intervention group. The median ECD decreased markedly from 2731 ± 18.3 cells/mm^2^ before the intervention to 1709 ± 20.2 cells/mm^2^ afterward (*p* < 0.001). The median ECD prior to transplantation was slightly higher in the lamellar group (2817 ± 38.1 cells/mm^2^) compared to the penetrating group (2717 ± 20.7 cells/mm^2^) *p* = 0.109. Postoperatively, however, the median ECD was lower in the lamellar group (1606 ± 37.5 cells/mm^2^) than in the penetrating group (1751 ± 22.8 cells/mm^2^) *p* = 0.014.

## 4. Discussion

Triple procedures have been shown to be an effective approach for restoring vision in patients with various corneal pathologies, including Fuchs’ endothelial dystrophy and bullous keratopathy [23,24]. This is evidenced by the significant improvement in BCVA observed following surgery. The relatively low rate of postoperative complications and re-graft procedures further supports the overall success of these surgical interventions [6]. The findings of this study provide insights into the demographic and clinical characteristics, procedural outcomes, and survival analysis of patients undergoing penetrating keratoplasty or posterior lamellar keratoplasty—DSAEK combined with cataract surgery.

The study revealed that rejection and decompensation rates were relatively low, with 86.7% of patients experiencing no rejection episodes. Only 3.3% had a single rejection episode or graft decompensation, while the remaining patients experienced multiple rejection events. Sridhar et al. (2000) found 72% graft clarity at last follow-up, slightly lower than our observed 86.7% rejection-free cases [25]. Javadi et al. (2013) reported even higher graft clarity (89.5%), aligning with our results [26].

In the literature, it was reported that lower rejection rates were observed after lamellar keratopasty compared to penetrating [27].

The analysis showed that lamellar transplant with cataract had a numerically higher median survival (47 months) compared to penetrating transplant with cataract (42 months). However, the difference is small and not statistically significant, as indicated by the hazard ratio and *p*-values. Additionally, while lamellar transplants initially displayed higher survival probabilities at earlier time points (e.g., 12 months), penetrating transplants had a slightly greater number of subjects at risk throughout the study period. Among the surgical techniques, PK was more commonly performed (72.1%) than posterior lamellar keratoplasty (27.9%). This aligns with the longstanding preference for PK in treating full-thickness corneal pathology and corneal donor shortage in Poland, although lamellar techniques have been gaining traction due to their benefits in preserving native corneal structure. The most common indication for a triple procedure was Fuchs endothelial dystrophy, followed by bullous keratopathy. This observation is concordant with the literature [6,11].

Age demonstrated a trend towards significance in influencing survival (HR = 0.98, *p* = 0.084), suggesting that younger patients may have slightly better survival outcomes. However, this did not reach statistical significance.

Cox regression analysis identified preoperative posterior corneal astigmatism as a significant risk factor for graft failure, associated with more than twice the risk of rejection or decompensation in both univariable (HR = 2.14, *p* = 0.006) and multivariable models (HR = 2.01, *p* = 0.014). K2 real demonstrated a trend toward significance (HR = 1.05, *p* = 0.060), suggesting a potential role in graft prognosis. K2 real refers to the steepest anterior corneal curvature, directly measured (not simulated) over a defined central corneal zone. Clinically, a higher or irregular steep corneal curvature reflects more advanced disease and biomechanical instability, which may increase the risk of poor healing, elevated postoperative astigmatism, and reduced graft survival. These findings underscore the predictive value of both K2 real and posterior astigmatism, supporting their consideration in preoperative risk assessment.

The studies indicate that the combined procedure provides significant advantages while maintaining safety and effectiveness comparable to sequential surgery. Terry et al. found no statistically significant difference in endothelial cell loss between eyes undergoing sequential surgery and those undergoing the triple procedure. Cazabon et al. (2010) also compared sequential versus combined PK and cataract surgery, reporting no significant difference in rejection rates, supporting our observation that surgical selection can be based on patient characteristics [28,29,30]. Nguyen et al. (2001) highlighted that blood–aqueous barrier (BAB) breakdown was significantly higher in triple procedures compared to PK alone, suggesting a potential impact on long-term rejection risks [31].

Sykakis et al. (2015) compared staged and combined endothelial keratoplasty procedures, finding comparable final visual outcomes despite a higher complication rate in combined procedures [8].

However, despite this increased postoperative response, studies have shown that it does not necessarily translate into higher rejection rates or compromised graft survival [10,32,33,34].

DSAEK resulted in better visual and refractive outcomes compared to PK. Although survival was statistically comparable, the quality of vision postoperatively favors lamellar approaches. Complication rates were acceptably low in both groups. The results of our study are in agreement with other researchers who compared the visual outcomes of lamellar and penetrating keratoplasty [7,35]. However, Patel et al. found no differences in BCVA between these two groups [36].

Central corneal thickness (CCT) was reported as a prognostic factor for graft survival in patients undergoing PK, particularly in high-risk cases, as demonstrated by McDonnell et al. [37]. However, in our study, CCT measured at 12 months postoperatively showed no significant association with transplant rejection or survival in the multivariable model. The earlier timepoint used in the McDonnell study may partly explain the differing results. Additionally, the literature on CCT in PK patients remains contradictory. The underlying disease appears to be the primary factor influencing postoperative CCT changes. Previous reports showed an increase in CCT in keratoconus, no significant change in Acanthamoeba keratitis, and a decrease in bullous keratopathy [38,39,40]. In our cohort, a decrease in CCT was observed following the penetrating triple procedure, although it was not statistically significant. Similar findings were reported by Machalińska et al. in lamellar keratoplasty at 12-month follow-up [41]. Moreover, Lombardo et al. demonstrated that thinner grafts in triple DSAEK procedures were associated with a hyperopic shift, highlighting a correlation between graft thickness and refractive outcomes [7,42,43]. The BCVA after the triple procedure is excellent and the data from this study indicate that it is comparable to that obtained when DSAEK surgery is performed alone [44]. Patients value the rapid visual recovery enabled by DSAEK surgery. By performing the triple procedure, surgeons further accelerate this recovery compared to sequential surgery while also minimizing the risks and costs associated with a second operation [10,29,45].

Triple DSAEK and triple DMEK procedures provide significantly improved refractive predictability due to the relative stability of mean corneal power, which is maintained by minimizing alterations to the recipient’s corneal anatomy [46,47]. However, regarding PK, it was shown that the deviation from target refraction was greater in patients undergoing simultaneous keratoplasty and cataract surgery compared to those who had sequential procedures [47]. Nevertheless, some studies do not support a significant difference in visual and refractive outcomes between the triple procedure and sequential surgery [10].

Astigmatism remained significantly higher after PK, along with corneal curvature (K1, K2, and Kmax), further highlighting the refractive challenges associated with this technique compared to lamellar approaches.

The high variability observed in PK patients suggests a more heterogeneous population, potentially reflecting differences in underlying corneal pathology or variability in surgical outcomes. This aligns with the findings of Das et al. (2006) [47], who emphasized that high refractive errors remain a challenge following PK, reinforcing our observations. In contrast, Schoenberg et al. (2015) reported generally favorable refractive outcomes after DMEK triple procedures, with a significant improvement in median best-corrected distance visual acuity [43]. Similarly, Oie and Nishida (2016) discussed recent advancements in triple procedures, further supporting its growing adoption and improved refractive predictability, particularly in endothelial keratoplasty [27]. These findings highlight the advantages of lamellar approaches in reducing refractive unpredictability while acknowledging the challenges associated with penetrating keratoplasty in achieving optimal postoperative refractive outcomes.

The observed decrease in median ECD from 2731 ± 18.3 to 1709 ± 20.2 cells/mm^2^ (*p* < 0.001) is consistent with the reported 30–50% endothelial loss following corneal transplantation, attributed to surgical trauma, tissue handling, and inflammation [48,49].

Although the median preoperative ECD was numerically higher in the DSAEK group (2817 ± 38.1 cells/mm^2^) compared to the PK group (2717 ± 20.7 cells/mm^2^), this difference was not statistically significant (*p* = 0.109). Interestingly, postoperative ECD was significantly lower in the DSAEK group (1606 ± 37.5 cells/mm^2^) than in the PK group (1751 ± 22.8 cells/mm^2^; *p* = 0.014), contrary to prior studies suggesting better ECD preservation with DSAEK [50,51]. This may be partly explained by the limited availability of postoperative ECD data.

In Poland, due to the shortage of corneal transplant tissues and the risk of tissue loss associated with the challenges of graft preparation in DMEK procedures, this method has long accounted for only a small percentage of performed transplants. Previous analyses of corneal transplants in Poland confirm that PK remained the dominant technique, primarily due to the limited availability of donor tissues and surgeons’ concerns regarding the technical difficulties and potential failure of DMEK [23]. However, in recent years, we have observed a growing trend in the number of DMEK procedures performed at our center. Advances in surgical techniques and improved access to grafts have facilitated the gradual increase in these procedures.

The findings of our study indicate significant differences in refractive outcomes between the two analyzed groups, with patients after PK exhibiting higher variability in both SE and astigmatism. The significantly greater mean astigmatism aligns with previous research showing that PK often results in higher postoperative astigmatism due to the condition and size of both the donor graft and the recipient corneal bed, the positioning and tension of sutures—which depend on the chosen suturing technique, the surgeon’s expertise, or the presence of synechiae [30]. The greater deviation from target refraction in this group supports existing evidence that penetrating keratoplasty is associated with increased refractive unpredictability compared to lamellar techniques.

The refractive outcomes following the newer triple procedure, which combines endothelial keratoplasty with cataract surgery, are markedly superior to those achieved with penetrating keratoplasty combined with cataract surgery [6,29,30,35]. Overall, these findings highlight the importance of tailoring surgical approaches to individual patient needs, balancing refractive stability with other clinical considerations. Future research should explore long-term functional outcomes and optimal strategies for managing postoperative astigmatism.

## 5. Limitations

This study provides valuable insights into the outcomes of triple procedures involving penetrating keratoplasty and Descemet stripping automated endothelial keratoplasty; however, several limitations must be acknowledged. The retrospective design introduced inherent biases, including potential selection bias and incomplete data capture. In particular, the absence of comprehensive postoperative endothelial cell density data limited the evaluation of long-term graft function, which is a key indicator of transplant success.

The extended duration of the study introduced variability in follow-up times, particularly among more recent DSAEK cases. Comparisons between PK and DSAEK were further constrained by differences in sample size, surgical technique, and baseline patient characteristics. As a single-center study, the generalizability of these findings may be limited.

Data completeness regarding corneal topography, visual acuity, and postoperative complications varied across cases, restricting the analysis of certain prognostic factors. Additionally, anterior segment optical coherence tomography imaging was only available for a subset of patients from the later study period (*n* = 317), limiting related analyses to this subgroup. Lastly, although principal component analysis was applied to address multicollinearity in the Cox regression model, the low events-per-variable ratio reduced the statistical power and reliability of the multivariable survival analysis. This is acknowledged as a key limitation.

## 6. Conclusions

Our findings have several clinical implications. The comparable survival rates between penetrating and lamellar transplants suggest that surgical selection can be tailored based on individual patient characteristics rather than concerns about long-term viability. The increased anterior astigmatism postoperatively highlights the need for improved refractive management post-transplantation, potentially through tailored suture techniques or adjunctive refractive procedures. PK triple procedures are highly successful anatomically, ensuring graft clarity and long-term viability. However, their functional outcomes remain limited, yielding only moderate visual improvement.

Future studies should focus on refining risk prediction models for transplant survival by incorporating additional factors such as inflammatory markers and long-term medication adherence. Additionally, exploring strategies to mitigate astigmatism could improve overall visual outcomes and quality of life for recipients.

## Figures and Tables

**Table 1:** Preoperative and postoperative best-corrected visual acuity (BCVA), spherical equivalent (SE) in the analyzed groups.

Parameter	Group 1	Group 2	*p*-Value
Preoperative Visual Acuity (Mean ± SD)	0.232 ± 0.123	0.290 ± 0.138	<0.0001
Postoperative Best-Corrected Visual Acuity (Mean ± SD)	0.372 ± 0.150	0.505 ± 0.200	<0.0001
Spherical Equivalent (SE) Mean ± SD	1.83 ± 4.39	1.02 ± 1.24	0.026
Astigmatism Mean ± SD	3.17 ± 2.02	1.42 ± 1.05	<0.0001

**Table 2:** Descriptive statistics of corneal topography. All keratometric values are in diopters (D); central corneal thickness in micrometers (µm).

Parameter	Lamellar Mean	Lamellar Median	Lamellar SD	Lamellar SE	Penetrating Mean	Penetrating Median	Penetrating SD	Penetrating SE	*p*-Value
CCT Before	647.97	633.0	232.05	19.54	705.79	688.0	148.74	22.68	0.126
CCT After	604.77	572.0	138.16	11.63	632.63	615.0	80.21	12.23	0.210
Anterior Corneal Astigmatism Before	6.51	3.67	6.78	0.57	1.59	1.48	0.76	0.12	<0.001
Anterior Corneal Astigmatism After	7.21	7.21	4.23	0.36	1.57	1.53	0.65	0.1	<0.001
Posterior Corneal Astigmatism Before	1.21	0.78	1.18	0.1	0.63	0.52	0.52	0.08	0.002
Posterior Corneal Astigmatism After	1.65	1.29	1.41	0.12	0.88	0.6	0.83	0.13	<0.001
Total (Real) Corneal Astigmatism Before	7.34	3.64	11.7	1.01	2.02	1.78	1.22	0.19	0.003
Total (Real) Corneal Astigmatism After	8.6	7.28	6.64	0.58	2.27	1.83	1.56	0.24	<0.001
K1 Anterior Before	47.07	44.18	10.39	0.88	43.89	44.3	2.14	0.33	0.048
K1 Posterior Before	−6.75	−6.07	1.75	0.15	−6.25	−6.11	1.03	0.16	0.077
K1 Posterior After	−6.87	−6.6	1.31	0.11	−6.83	−6.63	1.2	0.18	0.862
K1 Real Before	45.9	43.24	10.21	0.88	42.88	42.35	2.34	0.36	0.057
K1 Real After	39.05	38.12	9.86	0.86	42.33	42.13	2.8	0.43	0.033
K2 Anterior Before	53.77	47.6	13.59	1.14	45.28	45.48	2.41	0.37	<0.001
K2 Posterior Before	−7.96	−6.98	2.34	0.2	−6.72	−6.39	1.16	0.18	<0.001
K2 Posterior After	−8.38	−8.01	2.39	0.21	−7.19	−6.96	1.15	0.18	0.002
K2 Real Before	52.84	46.11	16.96	1.46	44.42	43.91	2.3	0.35	0.001
K2 Real After	47.35	45.95	10.44	0.91	43.67	43.72	2.49	0.38	0.024
K max Before	64.76	53.27	21.2	1.79	47.88	47.71	2.87	0.44	<0.001
K max After	64.13	56.12	19.95	1.68	45.9	46.47	6.82	1.04	<0.001

## Data Availability

Data supporting the findings are available from the corresponding author upon reasonable request.

## Abbreviations

The following abbreviations are used in this manuscript:

PK	Penetrating Keratoplasty
DSAEK	Descemet Stripping Automated Endothelial Keratoplasty
BCVA	Best-Corrected Visual Acuity
SE	Spherical Equivalent
CCT	Central Corneal Thickness
